# Supplementary material for: Novel Driver Strength Index highlights important cancer genes in TCGA PanCanAtlas patients
Source: PeerJ. 2022 Aug 11;10:e13860. doi: 10.7717/peerj.13860 (PMC9375969; doi:10.7717/peerj.13860)
Supplement: Supplemental Information 2 [file peerj-10-13860-s002.zip › COHORTS/genes plots/cohorts/2021_8_16_14_9_distribution_cohorts_males_STAD.pdf]

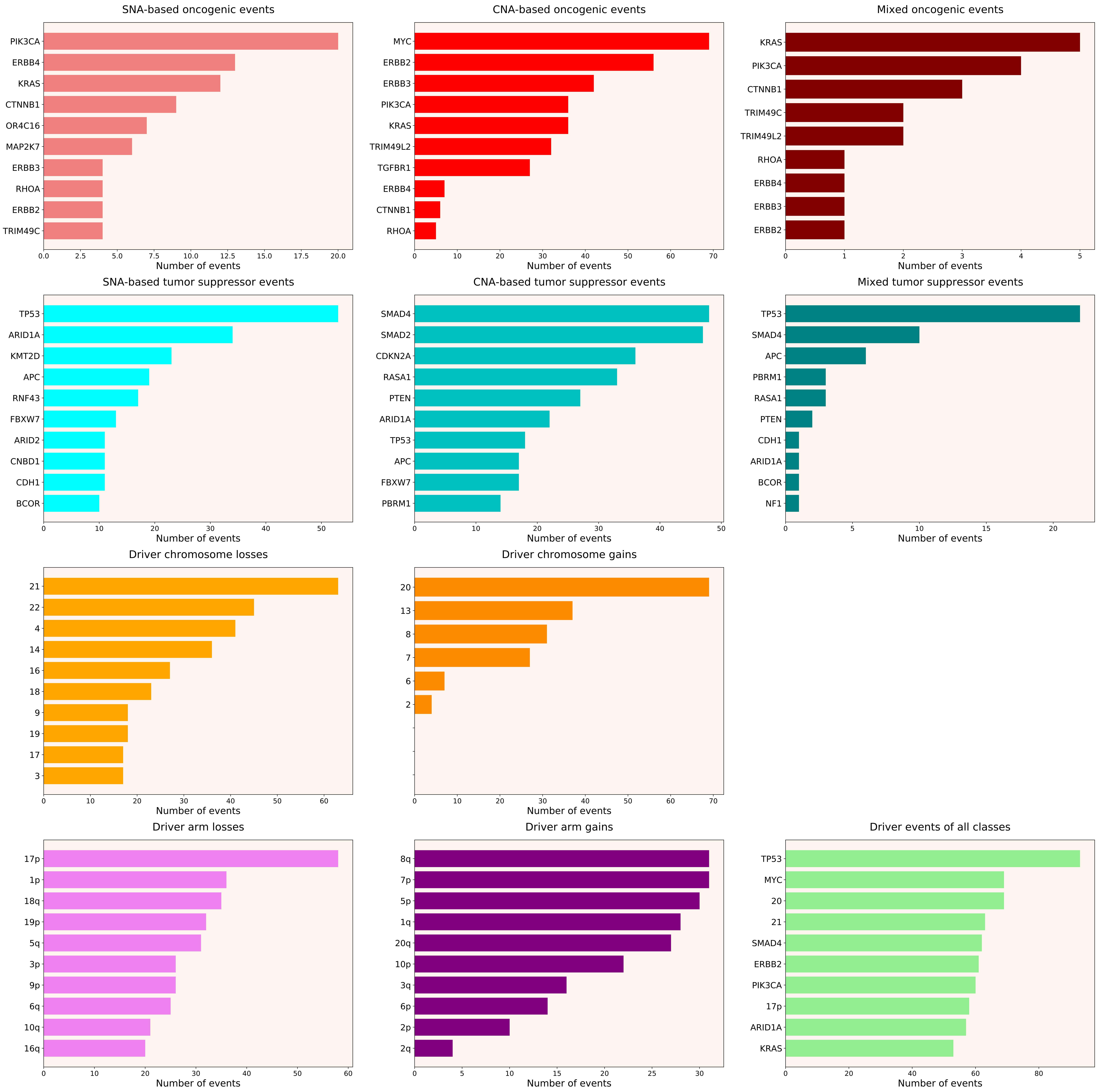
SNA-based oncogenic events
PIK3CA
ERBB4
KRAS
CTNNB1
OR4C16
MAP2K7
ERBB3
RHOA
ERBB2
TRIM49C
0.0 2.5 5.0 7.5 10.0 12.5 15.0 17.5 20.0
Number of events
CNA-based oncogenic events
MYC
ERBB2
ERBB3
PIK3CA
KRAS
TRIM49L2
TGFBR1
ERBB4
CTNNB1
RHOA
0 10 20 30 40 50 60 70
Number of events
Mixed oncogenic events
KRAS
PIK3CA
CTNNB1
TRIM49C
TRIM49L2
RHOA
ERBB4
ERBB3
ERBB2
0 1 2 3 4 5
Number of events
SNA-based tumor suppressor events
TP53
ARID1A
KMT2D
APC
RNF43
FBXW7
ARID2
CNBD1
CDH1
BCOR
0 10 20 30 40 50
Number of events
CNA-based tumor suppressor events
SMAD4
SMAD2
CDKN2A
RASA1
PTEN
ARID1A
TP53
APC
FBXW7
PBRM1
0 10 20 30 40 50
Number of events
Mixed tumor suppressor events
TP53
SMAD4
APC
PBRM1
RASA1
PTEN
CDH1
ARID1A
BCOR
NF1
0 5 10 15 20
Number of events
Driver chromosome losses
21
22
4
14
16
18
9
19
17
3
0 10 20 30 40 50 60
Number of events
Driver chromosome gains
20
13
8
7
6
2
0 10 20 30 40 50 60 70
Number of events
Driver arm losses
17p
1p
18q
19p
5q
3p
9p
6q
10q
16q
0 10 20 30 40 50 60
Number of events
Driver arm gains
8q
7p
5p
1q
20q
10p
3q
6p
2p
2q
0 5 10 15 20 25 30
Number of events
Driver events of all classes
TP53
MYC
20
21
SMAD4
ERBB2
PIK3CA
17p
ARID1A
KRAS
0 20 40 60 80
Number of events
